# Save the Babies

**Published:** 1873-01

**Authors:** 


SAVE THE BABIES.—On good authority, it is promulgated abroad that two drops of turpentine oil in a little milk is a complete antidote to phosphorus poison. Children not unfrequently bite off the charged end of phosphoric matches and swallow them. It is stated that a girl was recently saved in England by this newly-discovered remedy, who had actually eight match ends in her stomach.—*Scientific Press.*

---

THE mere lapse of years is not life. To eat, and drink, and sleep—to be exposed to darkness and light—to pace round in the mill of habit, and turn thought into an implement of trade—this is not life. Knowledge, truth, love, beauty, goodness, faith, alone can give vitality to the mechanism of existence.

